# Elevation of Serum Transaminase Levels Due to Favipiravir Use in the Treatment of COVID-19

**DOI:** 10.7759/cureus.18166

**Published:** 2021-09-21

**Authors:** Mehmet Bayram, Ozgur Yildirim, Raye Sevra Ozmen, Beyza Soylu, Ahmet Said Dundar, Ali Riza Koksal, Murat Akarsu, Abdulbaki Kumbasar, Omur Tabak

**Affiliations:** 1 Gastroenterology and Hepatology, Health Sciences University Kanuni Sultan Süleyman Training and Research Hospital, Istanbul, TUR; 2 Department of Internal Medicine, Health Sciences University Kanuni Sultan Süleyman Training and Research Hospital, Istanbul, TUR; 3 Gastroenterology and Hepatology, Tulane University School of Medicine, New Orleans, USA; 4 Department of Internal Medicine, Health Sciences University Kanuni Sultan Suleyman Training and Research Hospital, Istanbul, TUR

**Keywords:** icu admission, mortality, elevated transaminases, covid-19, favipiravir

## Abstract

Background and aims: Favipiravir is a ribonucleic acid (RNA)-dependent RNA polymerase (RdRP) inhibitor antiviral agent used in the treatment of coronavirus disease-2019 (COVID-19). In this study, we investigated the changes in serum transaminase levels of patients and the relationship between serum transaminase elevation with mortality in patients who were hospitalized with the diagnosis of COVID-19 and received favipiravir treatment.

Materials and methods: 454 patients who received favipiravir and 113 patients who did not receive favipiravir were evaluated. Serum transaminase levels of the patients were compared at baseline and after five days of treatment, and the relationship between serum transaminase elevation and mortality was investigated.

Results: No significant aspartate aminotransferase (AST) or alanine aminotransferase (ALT) elevation was detected due to favipiravir treatment. AST elevation was found, respectively, as 133 (29.3%), 32 (28.3%) (p=0.100), ALT elevation as 112 (24.7%), 35 (29.3%) (p=0.100) in the groups receiving and not receiving favipiravir. High AST level was found as a risk factor for mortality in all patient groups (p=0.008).

Conclusions: There was no statistically significant elevation in serum transaminase levels due to favipiravir use in patients hospitalized for COVID-19. A high level of AST is a significant risk factor to show mortality and intensive care unit (ICU) admission in patients with COVID-19.

## Introduction

Coronavirus is a non-segmented single-stranded ribonucleic acid (RNA) virus, and it has six species that cause disease in humans [1]. Among these, SARS-CoV-2 (severe acute respiratory syndrome coronavirus 2) was detected for the first time in China in December 2019 and subsequently spread to more than 100 million people in more than 200 countries, causing a global pandemic. World Health Organization (WHO) declared an emergency in January 2020 and a pandemic on March 11, 2020, the disease name was determined as coronavirus disease-2019 (COVID-19). COVID-19 is a disease that can progress in a broad spectrum from asymptomatic mild illness to severe lung disease [2,3]. Hepatic injury in COVID-19 may develop due to the direct cytopathic effect of the virus, uncontrolled immune response, hypoxia secondary to pneumonia, and the drugs used in treatment [4,5]. In the literature, the rate of elevation in serum transaminase levels of patients hospitalized for COVID-19 was detected to be 37.5% [6]. Also, elevation in serum transaminase levels in COVID-19 patients has been detected to be associated with increased mortality in the literature [7,8].

Favipiravir is an antiviral agent developed in Japan for the treatment of drug-resistant influenza [9]. RNA-dependent RNA polymerase (RdRP) plays an important role in coronavirus replication. Favipiravir shows its effect by inhibiting RdRP [9]. The effect of the agent on SARS-CoV-2 has been demonstrated in vitro [10]. In early clinical studies, it was detected that the drug reduced viral load in patients and provided clinical and radiological improvement [11,12]. Diarrhea, neutropenia and elevation of serum transaminase levels are the most common side effects of the drug. In a review including patients using favipiravir in the treatment of influenza and COVID-19, elevations in serum transaminase levels were detected in 2% of patients after drug use [13]. With the use of favipiravir in COVID-19, an elevation in serum transaminase levels was detected in 8.6% of the patients [14]. In our study, we investigated the elevation of serum transaminase levels and the clinical significance of this situation in groups of patients who received and did not receive favipiravir treatment while in hospital with the diagnosis of COVID-19.

## Materials and methods

Study design and data collection

The study was conducted retrospectively using the medical records of inpatients with positive COVID-19 real-time polymerase chain reaction (RT-PCR) test with signs of COVID-19 pneumonia on thorax CT (computed tomography) between April 1, 2020, and December 31, 2020, at Kanuni Sultan Suleyman Training and Research Hospital. In the study, 454 patients who received favipiravir and 113 patients who did not receive favipiravir among the patients who were hospitalized with normal aspartate aminotransferase (AST) and alanine aminotransferase (ALT) levels (baseline) were evaluated. Patients in the moderate COVID-19 clinical category (room air SpO_2_< 95%, shortness of breath, respiratory rate>24, fatigue, and heart rate>110) were included in the study [15]. According to the COVID-19 treatment protocol of the Turkish Health Ministry, during hospitalization, the patients were administered favipiravir treatment for five days as 3200 mg on the first day and 1200 mg after that. In addition, subcutaneous enoxaparin treatment was given to the patients. Complete blood count (CBC), AST, ALT, C-reactive protein (CRP), and albumin levels at the beginning of hospitalization of the patients and control serum transaminase levels after five days of treatment were recorded and analyzed. CBC parameters were determined by a hematology analyzer (Mindray BC-6800Plus, Mindray, Shenzen, China). Biochemical parameters were measured by the Roche Cobas Integra 800 (Roche Diagnostic Limited, Switzerland) device. Intensive care unit (ICU) admission and mortality due to the disease were recorded. Patients with viral hepatitis, autoimmune hepatitis, and other chronic liver diseases and those with high basal transaminase levels, and those who started on favipiravir with another drug that could cause transaminase elevation were excluded from the evaluation. In addition, patients with diseases and comorbidities that may cause an increase in serum transaminase levels were not included in the study. After five days of treatment, patients with AST and ALT levels>40 U/L in control tests were considered to have elevated serum transaminase levels. The difference between serum transaminase levels and the development of elevated serum transaminases in the groups that received and did not receive favipiravir was analyzed. Also, the relationship between elevation of serum transaminases with the ICU admission and mortality in all patients was investigated. This study was approved by the Local Ethical Committee of Kanuni Sultan Süleyman Training and Research Hospital (approval number: KAEK /2021.01.18) and was conducted following the Declaration of Helsinki.

Statistical analysis

All analyses were performed using SPSS version 21.0 for Windows (IBM Inc., Chicago, Illinois, USA). The variables were investigated using visual (histograms) and analytical methods (Kolmogorov-Smirnov/Shapiro-Wilk tests) to determine whether or not they were normally distributed. The Chi-square test was used for comparisons of categorical variables. Ordinal variables and continuous variables that do not have normal distribution were compared by the Mann-Whitney U test. The Student t-test was used to evaluate differences between the two groups in normally distributed continuous variables. For multivariate analysis, the possible factors identified by univariate analysis or recent studies were further entered into the logistic regression analysis to determine independent predictors of ICU admission and mortality. Variables that did not make a significant contribution were excluded from the model using the backward likelihood ratio stepwise method. Hosmer-Lemeshow goodness-of-fit statistics were used to assess the model. A value of p<0.05 (two-sided) was considered statistically significant. Values are presented using means ± standard deviations for normally distributed and medians and first and third quartiles in the brackets for the non-normally distributed variables.

## Results

**Figure 1:**
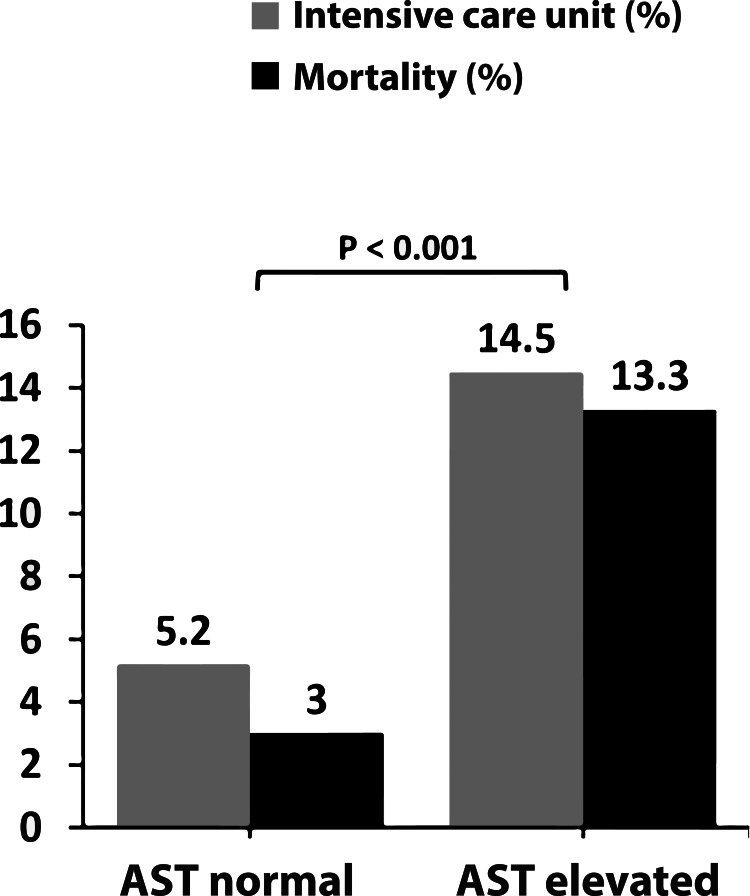
Relationship between AST elevation with mortality and ICU admission Mortality and ICU admission rates were significantly higher in AST elevated group than the AST normal group (p< 0.001). ICU: intensive care unit, AST: aspartate aminotransferase.

In the study, 454 patients who received favipiravir treatment and 113 patients who did not were included. AST elevation was found, respectively, as 133 (29.3%), 32 (28.3%; p=0.100), ALT elevation as 112 (24.7%), 35 (29.3%; p=0.100) in the groups receiving and not receiving favipiravir. In the group that received favipiravir, serum AST in 26 (%5,7) patients and serum ALT in 33 (%7,2) patients elevated more than two times the upper limit. Besides that, in the group that did not receive favipiravir, serum AST in four (%3,5) patients and serum ALT in six (%5,3) patients elevated more than two times the upper limit. There was no statistically significant difference between the two groups in terms of serum transaminase elevation rates. None of the patients developed severe serum transaminase elevation (more than five times the upper limit of normal) and liver failure. There was no statistically significant difference in mortality and need for ICU between the patient groups who received and did not receive favipiravir (p=0.99, p=0.92, respectively; Figure 1).

**Figure 2:**
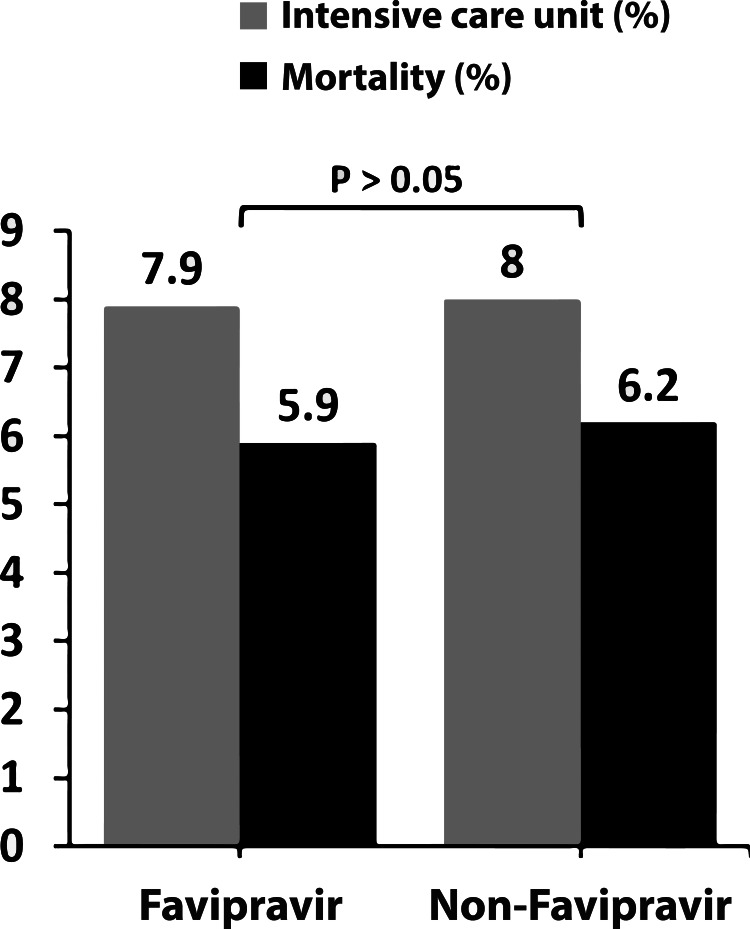
Mortality and ICU admission rates in receiving and not receiving favipiravir groups There was no statistically significant difference between patients who are treated with or without favipiravir in terms of mortality and ICU rates (p>0.05).

Age, gender, hematological and biochemical profile, demographic characteristics, ICU admission, and mortality rates in the patient groups are presented in Table 1. A statistically significant difference was found in the levels of CRP, albumin, and lymphocyte count between the patient groups who received and did not receive favipiravir (Table 1). ICU admission and mortality rates are presented in Table 2 when patients are grouped as AST and ALT elevated and non-elevated. There was a statistically significant difference between AST elevated and AST normal groups in terms of mortality and ICU admission rates (p<0.001; Figure 2). There was no statistically significant difference between ALT elevated and ALT normal groups in terms of ICU admission and mortality (Table 2). In univariate and multivariate logistic regression analysis, age, CRP, platelet count, and AST were determined as risk factors for mortality (Table 3). For ICU admission, age, CRP, platelet count, and AST were risk factors in univariate analysis, whereas age, CRP, and platelet count were found as independent risk factors in multivariate analysis (Table 4).

**Table 1:** The main clinical and biochemical characteristics of the patients received and not received favipiravir Values are presented using means ± standard deviations for normally distributed and medians and first and third quartiles in the brackets for the non-normally distributed variables; ALT: alanine aminotransferase; AST: aspartate aminotransferase; CRP: c-reactive protein; WBC: white blood cell; ICU: intensive care unit, SpO_2_: peripheral capillary oxygen saturation.

	Patients received favipiravir (n: 454)	Patients who not received favipiravir (n: 113)	P-value
Age	63.0 ± 15.9	63.8 ±11.8	0.590
Male gender (n %)	227 (%50)	68 (%58.8)	0.053
AST (baseline) U/L	26 (20-31)	25 (21-32)	0.960
ALT (baseline) U/L	20 (16-26)	20 (15-30)	0.650
AST (after 5 days of treatment) U/L	30 (21-44)	26 (19-48)	0.160
ALT (after 5 days of treatment) U/L	24 (15-40)	23 (16-45)	0.800
Albumin (mg/dL)	35.1 ± 4.6	36.8 ± 5.1	0.003
CRP (mg/dL)	72 (26-115)	33 (14-81)	0.001
WBC (10³/µl)	6.6 (4.9-9.1)	6.5 (5.1-7.8)	0.300
Lymphocyte (10³/µl)	1.2 (0.8-1.6)	1.4 (1-1.8)	0.001
AST elevation (n %)	133 (%29.3)	32 (%28.3)	0.830
ALT elevation (n %)	112 (%24.7)	35 (%29.3)	0.170
SpO_2_ (%)	93(90-94)	93 (91-94)	0.167
Respiratory rate (n/minute)	27(26-30)	27 (26-29)	0.681
ICU (n %)	36 (%7.9)	9 (%8)	0.990
Mortality (n %)	27 (%5.9)	7 (%6.8)	0.920

**Table 2:** Mortality and ICU admission in patient groups with and without serum transaminase elevation ALT: alanine aminotransferase; AST: aspartate aminotransferase; ICU: intensive care unit.

	AST elevated n:165 (%29.1)	AST normal n:402 (%70.9)	P-value	ALT elevated n:147 (%25.9)	ALT normal n:420 (%74.1)	P-value
ICU (n %)	24(%14.5)	21(%5.2)	0.001	6 (%4.1)	39(%9.3)	0.06
Mortality (n %)	22(%13.3)	12(%3)	0.001	5(%3.4)	29(%6.9)	0.12

**Table 3:** Predictors for mortality in hospitalized patients with COVID infection AST: aspartate aminotransferase; CRP: c-reactive protein.

	Univariate Analysis				Multivariate Analysis			
		%95 confidence interval				%95 confidence interval		
	OR	Lower bound	Upper bound	p-values	OR	Lower bound	Upper bound	p-values
Age	1.068	1.037	1.100	0.001	1.068	1.037	1.100	0.001
CRP	1.006	1.002	1.010	0.001	1.008	1.003	1.012	0.001
Platelet	0.994	0.989	0.999	0.016	0.994	0.989	0.999	0.014
AST	1.059	1.015	1.102	0.008	1.051	1.006	1.097	0.026

**Table 4:** Predictors for ICU admission in hospitalized patients with COVID infection AST: aspartate aminotransferase; CRP: c-reactive protein.

	Univariate analysis					Multivariate analysis					
		%95 Confidence Interval					%95 Confidence Interval				
	OR	Lower bound	Upper bound		p values	OR	Lower bound		Upper bound	p values	
Age	1.056	1.031	1.083	0,001		1.057	1.033	1.083			0.001
CRP	1.006	1.002	1.009	0.011		1.006	1.002	1.010			0.002
Platelet	0.995	0.991	0.999	0.012		0.995	0.990	0.999			0.009
AST	1.013	1.003	1.024	0.011		1.011	1.000	1.023			0.058

## Discussion

When the literature is reviewed, this study is the first one that gives priority to the evaluation of the relationship between the use of favipiravir and elevated serum transaminases in the treatment of COVID-19 patients. In our study, no significant difference was found in the development of serum transaminase elevation between COVID-19 patients used and did not use favipiravir. In addition, in our study, AST elevation was found to be a significant risk factor for mortality and ICU admission in COVID-19 patients.

Favipiravir is a prodrug that converts to the active form by intracellular phosphorylation [12]. Metabolized by aldehyde oxidase and partially xanthine oxidase in the liver, it turns into an inactive metabolite and is excreted by the renal route [16]. Metabolism of a drug from the liver affects its hepatotoxicity potential [17]. Serum transaminase level elevation in favipiravir use has been reported as 8.6% in a clinical trial and as 2% in a systematic review [13,14]. In two different studies, the prevalence of COVID 19-induced elevation of transaminases was found to be 21-46% for AST and 19-35% for ALT [2,18]. Similar to these results, in our study, COVID-related transaminase elevation was observed in 25% and 27% for AST and ALT, respectively. In addition, this elevation was less than five times the upper limit of normal in all of our patients and none of the patients developed severe transaminase elevation and liver failure. These results were also compatible with the literature data [18]. Current data show that COVID-19 patients with severe clinical findings develop irregular cytokine release, also known as "cytokine storm." Cytokine storm causes an extreme inflammatory and immune response that leads to acute respiratory distress syndrome (ARDS), pulmonary edema, and multi-organ failure. That means the overactive and untreated immune response in COVID-19 can be mortal [19]. Serum transaminase elevation in COVID-19 is associated with the severity of the disease and increased inflammatory markers [18]. Hepatocellular injury can be associated with the direct cytopathic effect of the virus, hyperinflammatory state, thrombotic microangiopathy, and hypoxia secondary to pneumonia [4,5,18]. Increased viral load in SARS-CoV-2 is associated with increased IL-6 level, cytokine storm, and poor prognosis [20,21]. Favipiravir is a potent inhibitor of viral proliferation. It shortens the time of viral clearance, possibly decreases cytokine production, and protects the lung from inflammatory damage [16,22,23]. Although it is known that favipiravir use causes elevated transaminase levels, no significant difference was observed in the elevation of transaminase levels between the patient groups using and not using the drug.

Serum transaminase elevations are indicators of hepatocellular damage and are detected in 8% of the population [24]. While ALT elevation is observed in clinical pictures such as hepatitis and drug-induced toxicity, AST elevation may also develop secondary to non-hepatic causes such as alcohol-induced hepatitis, hemolysis, myopathy, and exercise [24]. Aminotransferase elevation may be caused by myositis, and in COVID-19, the elevation of creatine kinase was detected in patients at a rate of 14% [2]. When considering along with the literature data, in our study, AST elevation in COVID-19 patients was evaluated as secondary to disease-related inflammatory response, while ALT elevation was evaluated as secondary to drug-related hepatotoxicity [25]. In our study, a significant relationship was found between AST elevation with mortality and ICU admission. Conversely, the mortality and ICU admission rates did not change in patients with ALT elevation. This contrast between the results also supports the idea mentioned above. CRP and IL-6 levels were detected at the same levels in patients with COVID-19 pneumonia and non-COVID pneumonia [26]. However, higher serum transaminase levels were detected in patients with grade III COVID-19 pneumonia than non-COVID pneumonia patients [27]. In our study, we detected elevated AST levels as a risk factor for mortality due to COVID-19 disease. On the other hand, for serum ALT level, a borderline inverse correlation was detected for the ICU admission, but there was no correlation with mortality. Both the lack of significant elevation in serum transaminases due to favipiravir use and the strong relation of AST value with ICU and mortality compared to ALT suggests that the elevation of serum transaminases in COVID-19 may be primarily due to the hyperinflammatory condition and cytopathic effect of the virus. There is a need for multicenter studies with larger patient numbers on the elevation of transaminases in favipiravir treatment in COVID-19.

The limitations of the study are its retrospective nature and the low number of patients who did not receive favipiravir. However, the fact that the group that did not receive favipiravir had better inflammatory and mortality-related markers such as CRP and lymphocyte compared to the other group may be considered as a limitation. This may be the result of the use of favipiravir in the treatment of more severe COVID-19 patients due to difficulties in drug supply in the early stages of the epidemic.

## Conclusions

In conclusion, there was no statistically significant elevation in serum transaminase levels due to favipiravir use in patients who were hospitalized for COVID-19. According to our study, the use of Favipiravir in the treatment of COVID-19 seems safe in terms of the development of serum transaminase elevation in patients. Additionally, the elevation of AST levels seems to be a significant risk factor for mortality and ICU admission in patients with COVID-19. Patients with elevated AST levels during hospitalization should be followed up more closely by clinicians in terms of the prognosis of the disease. Further multicenter studies with larger patient samples are needed on this subject.
